# Structural insights into mode of actions of novel natural Mycobacterium protein tyrosine phosphatase B inhibitors

**DOI:** 10.1186/1471-2164-15-S1-S3

**Published:** 2014-01-24

**Authors:** Jaspreet Kaur Dhanjal, Sonam Grover, Sudhanshu Sharma, Ajeet Kumar Singh, Abhinav Grover

**Affiliations:** School of Biotechnology, Jawaharlal Nehru University, New Delhi, 110067 India; Department of Biotechnology, Delhi Technological University, New Delhi, 110042 India; School of Life Sciences, Jawaharlal Nehru University, New Delhi, 110067 India

**Keywords:** QSAR, PTPB, Tuberculosis, Virtual screening, Tyrosine phosphatase, Inhibitor

## Abstract

**Background:**

Tuberculosis has become a major health problem being the second leading cause of death worldwide. *Mycobacterium tuberculosis* secretes a virulence factor, protein tyrosine phosphatase B (mPTPB) in the cytoplasm of host macrophage which suppresses its natural innate immune response and helps the pathogen survive and proliferate in the phagosome. The present study aims at indentifying potent inhibitors of mPTPB by using computational approaches of ligand based molecular modeling and docking studies.

**Results:**

A 3D QSAR model was developed using a set of benzofuran salicylic acid based mPTPB inhibitors with experimentally known IC_50_ values. The model was generated using the statistical method of principle component regression analysis in combination with step wise forward variable selection algorithm. It was observed that steric and hydrophobic descriptors positively contribute towards the inhibitory activity of the ligands. The developed model had a robust internal as well as external predictive power as indicated by the q^2^ value of 0.8920 and predicted r^2^ value of 0.8006 respectively. Hence, the generated model was used to screen a large set of naturally occurring chemical compounds and predict their biological activity to identify more potent natural compounds targeting mPTPB. The two top potential hits (with pIC_50_ value of 1.459 and 1.677 respectively) had a similar interaction pattern as that of the most potent compound (pIC_50_ = 1.42) of the congeneric series.

**Conclusion:**

The contour plot provided a better understanding of the relationship between structural features of substituted benzofuran salicylic acid derivatives and their activities which would facilitate design of novel mPTPB inhibitors. The QSAR modeling was used to obtain an equation, correlating the important steric and hydrophobic descriptors with the pIC_50_ value. Thus, we report two natural compounds of inhibitory nature active against mPTPB enzyme of *Mycobacterium tuberculosis*. These inhibitors have the potential to evolve as lead molecules in the development of drugs for the treatment of tuberculosis.

**Electronic supplementary material:**

The online version of this article (doi:10.1186/1471-2164-15-S1-S3) contains supplementary material, which is available to authorized users.

## Background

Tuberculosis (TB) is an infectious disease caused by *Mycobacterium tuberculosis (Mtb)*. It has become a major health problem being the second leading cause of death worldwide, after human immunodeficiency virus (HIV). According to the Global Tuberculosis Report, 2012 by World Health Organization, 8.7 million new cases of TB, 13% of which were co-infected with HIV and 1.4 million deaths from TB were estimated in 2011. TB is most prevalent in Asia and Africa with India and China alone accounting for about 40% of the global cases [1].

*Mtb* survives as an intracellular pathogen and replicates in the macrophages of its host organism. It disrupts the normal biochemical pathway of the phagosomes involved in defense against intracellular pathogens by phosphorylation or dephosphorylation of the host's proteins. A variety of cellular functions like proliferation, migration, apoptosis, immune response etc. require post translational modification of proteins by the process of tyrosine phosphorylation. In normal physiological conditions a balance is maintained between the activity of protein tyrosine kinases (PTKs) and protein tyrosine phosphatases (PTPs). Impairment of this controlled regulation may lead to anomalous tyrosine phosphorylation, which is believed to be responsible for many human diseases like cancer, diabetes and auto immune disorders among others. Thus, PTPs and PTKs are important targets for many diseases with high therapeutic value [2–5]. *Mtb* secretes a virulence factor, protein tyrosine phosphatase B (mPTPB) in the cytoplasm of host macrophage which suppresses the natural innate immune response of the phagosome against the TB infection by blocking the ERK1/2 and p38 mediated IL-6 B production and preventing host cell apoptosis by activating the Akt pathway [6, 7]. This prevents the phagosome from maturating into a phagolysosome for the destruction of invaded pathogen. To investigate the role of PTPB in pathogenesis of *Mtb*, a mutant strain of PTPB was created and the ability of the parent and the mutant strain to survive in the host macrophages was compared. In this experiment, it was found that the disruption of mPTPB gene resulted in 70-fold reduction in the bacterial load in the spleen of guinea pigs. Complementary strain, obtained after reintroducing the gene into the mutant strain, regained the ability to infect the guinea pigs at rates comparable to the parent strain [8]. Beresford et al. also studied the growth of mycobacteria in resting macrophages in order to mimic the infection in a susceptible host (where IFNγ activation may be impaired). Their study showed that in the absence of inhibitors of mPTPB, intracellular growth of mycobacterium increased. However when treated with a potent inhibitor, the intracellular mycobacterial growth decreased substantially [9]. All these studies suggest that mPTPB is a potential target against which inhibitors can be designed to develop new and effective anti-tuberculosis agents.

Today many drugs are available for clinical use to treat TB, but the current treatment lasts for six to nine months. During the course of treatment, the pathogen develops resistance against these drugs which results in Multi-Drug resistant Tuberculosis (MDR TB) and eventually lead to untreatable extensively drug resistant Tuberculosis (XDR TB) [10]. To overcome the problem of growing drug resistance, identification of new targets which are essential for survival and replication of the pathogen has become an urgent need. For the purpose of finding drugs against novel targets we require fast and reliable computational techniques for cost-effective evaluation of large virtual databases of chemical compounds in order to identify a limited set of candidates which can be synthesized and examined experimentally for their biological activity. Quantitative structure activity relationship (QSAR) is a powerful approach being used to establish a correlation between the physiochemical properties of the chemical compounds and their biological activity to obtain a reliable statistical model. This model serves as a valuable tool for the design of new chemical entities and to predict their activity. The QSAR model so developed facilitates identification of promising lead candidates, thus decreasing the number of compounds required to be synthesized and tested *in vitro* [11].

Zhou B *et al*., reported a benzofuran salicylic acid-based mPTPB inhibitor (I-A09) which showed modest potency and selectivity [6]. But the inhibitor was not effective for therapeutic clinical use. The chemistry-oriented approach was used to modify the core structure of I-A09 to obtain a highly potent and selective mPTPB inhibitor which also showed considerably good *in vivo* efficacy [2]. Additional file 1 mentions benzofuran salicylic acid derived compound series so developed along with their IC_50_ values. We have used this compound series containing 18 compounds for building the 3D-QSAR model and to identify the molecular features essential for effective interaction between the inhibitors and the active cleft of the mPTPB enzyme. The model thus generated using the same series of representative inhibitors was then used to predict the activity of a large dataset of natural compounds. The compounds whose predicted biological activity was greater than the most potent inhibitor of the congeneric series were then analyzed using *in silico* docking studies to elucidate their mode of interaction with the mycobacterium phosphatase.

## Materials and methods

### Data set

A data set consisting of 18 novel inhibitors of mPTPB derived from 6-hydroxy-benzofuran-5-carboxylic acid scaffold was taken from a previously reported study [2]. These inhibitors were highly selective for mPTPB over all other PTPBs which were examined. The reported biological activity data (IC_50_ values in µM) for these inhibitors was converted into logarithmic scale (pIC_50_) to be used for QSAR study.

### Molecular modeling study

**Figure 1:**
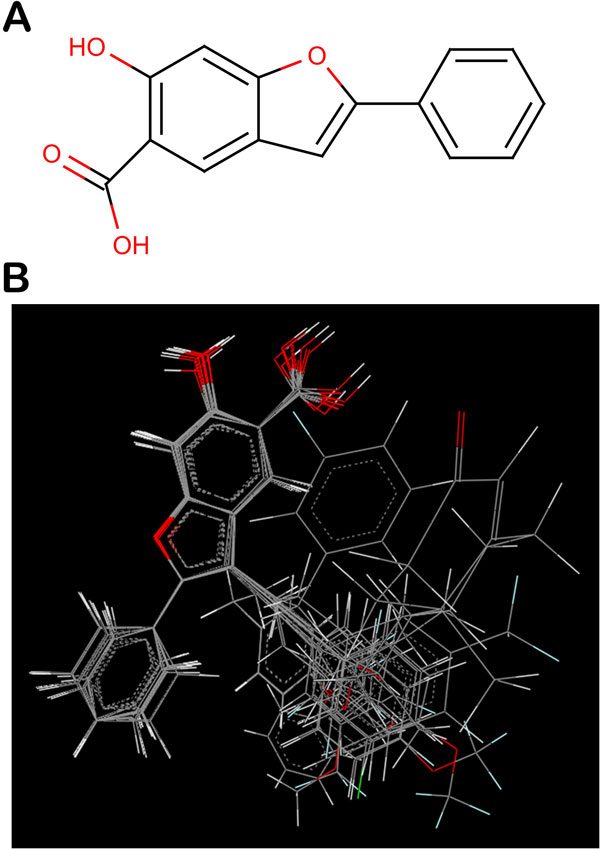
**(a) Structure of template used for template based alignment of optimized molecules (b) 3D alignment of optimized mPTPB inhibitors**.

The 2D structures were sketched using VlifeEngine of VLife MDS and then converted to 3D form. The 3D structures so obtained were optimized to attain a stable conformation with minimum energy using force field batch minimization platform of VlifeEngine. Merck Molecular Force Field (MMFF) and Gasteiger charges were used with maximum number of cycles as 10000, convergence criteria (root mean square gradient) as 0.01 and dielectric constant (for vaccum) as 1.0. A structure common to all 18 inhibitors was deduced and used as template (Figure 1a) to align all the geometry optimized mPTPB inhibitors. Alignment of all the inhibitors to the template molecule taking compound 10 (comp10) as the reference molecule is shown in Figure 1b. The whole study was performed on Intel ^®^ Xeon (R) CPU E31230 @ 3.20 GHz with 8.00 GB RAM using Vlife MDS, Molecular Design Suite, version 4.3, supplied by Vlife Sciences, Pune, India [12].

### Computation of values for descriptors and data selection for training and test set

A molecular field was computed for a grid of points in space around the aligned molecules using Merck molecular force field. Descriptors representing hydrophobic, electrostatic and steric energies between the atoms of the aligned molecules and a methyl probe with +1 charge placed at each lattice point of the grid were computed. These molecular descriptors describe how each of the inhibitory molecules binds to the target in its active site. For the external validation of the model, the data set was divided into training and test set using the approach of random selection to avoid any kind of bias. The training set (75% of the total molecules in the data set) with known biological activity was used to generate the 3D QSAR model. The test set, compounds of which were not included for building the model, was used to challenge the generated model to assess its predictive effectiveness.

### 3D QSAR model building

The model was generated using statistical method of principle component regression analysis (PCA) in conjunction with stepwise forward variable selection algorithm. pIC_50_ value was used as dependent variable and the descriptors as independent variables. Software generates a large number of molecular descriptors that can be used for the QSAR study. Because of this huge data, the choice of selection of appropriate descriptors having a considerable role in governing the biological activity of interest becomes difficult. Thus, the success of QSAR model greatly depends on the statistical method being employed for the model generation. PCA method is used when the number of molecular descriptors is much more than the number of observations in the system. It carefully excludes the group of variables with high internal correlation. It efficiently reduces the number of independent variables to be used in the QSAR model by removing all possible redundancy and limiting the variables with descriptor values to a smaller set of uncorrelated variables [13]. Various parameters were set for the execution of stepwise principle component regression analysis. The cross correlation limit was set as 0.5, maximum number of variable in final equation as 2 (n/5, where n is number of compounds in training set), term selection criteria as r^2^, variance cut-off as 0 and scaling as auto scaling.

### Validation of the 3D QSAR model

To establish a QSAR model two types of validations are required - internal and external. For internal validation leave-one-out cross validation method was used. In this method one observation was taken as validation data and the rest of the observations made up the training set. The coefficients of QSAR model were estimated using this new training set which were then used for predicting the activity of the test compound. The procedure was repeated until all the compounds had once served as a test compound. The predictive ability of the model was then assessed using the cross validated r^2^ and q^2^ [13]. External validation was done by predicting the activities of the compounds of the test set which were not used for model generation.

### Prediction of biological activity of a large data set of natural compounds using the generated 3D QSAR model

A data set consisting of 1,69,109 natural compounds by 10 different suppliers was obtained from ZINC database [14] in SMILES format. The pIC_50_ values were predicted for these natural compounds using the generic prediction platform of VlifeMDS. The prediction was done based on the QSAR model generated using the congeneric series consisting of 18 mPTPB inhibitors. The most potent compound in this series had a pIC_50_ value of 1.42. So the natural compounds with predicted activity above this threshold were selected for further analysis as they could prove to be more potent and selective novel candidates to be used as mPTPB inhibitors.

### Protein and ligand preparation for docking studies

The crystal structure of protein tyrosine phosphatase B of *Mtb* origin was obtained from Protein Data Bank [PDB ID: 1YWF] [15]. The protein structure was pre-processed by removing water molecules and all non-bonded heteroatoms using Accelyrs Viewerlite 5.0 [16]. This processed protein was further prepared using Schrödinger's protein preparation wizard [17]. Hydrogen were added and optimized to the structure. In further preparation steps bad contacts were removed, bond lengths were optimized, disulfide bonds were created, protein terminals were capped and selenomethionine residues were converted to methionine. The missing residues were fixed manually. The natural compounds with predictive pIC_50_ values above 1.42 were prepared for docking studies to study their mode of interactions with mPTPB. LigPrep's ligand preparation protocol was used to prepare these natural compounds. It generated different tautomeric, stereochemical and ionization variants of the small molecules along with energy minimization and flexible filtering.

A grid was generated at the active site of the prepared protein structure using the Glide docking module of Schrödinger [18]. The active site of PTPs lies in the P loop motif. CysX_5_Arg defines the consensus sequence of this loop. Catalytic Arg acts as a general acid in the reaction mechanism. Presence of histidine just before the active site cysteine makes it a better nucleophile. Therefore, residues His 160-Arg 166 constitute the active site of mPTPB [19]. Prepared natural compounds were subjected to docking using Glide's extra precision docking protocol. The two top scoring compounds were investigated to study their molecular interactions with the protein molecule. The hydrophobic interactions and H-bonds were calculated using the Ligplot program [20]. H-bonds were taken into consideration when the distance between acceptor-donor atoms was less than 3.3 Å, with maximum hydrogen-acceptor atom distance of 2.7Å and acceptor-H-donor angle greater than 90°.

## Results and discussion

### QSAR molecular modeling

QSAR study requires ligands with experimentally measured values of the desired biological activity. The ligands should ideally be a part of a congeneric series but should also possess adequate chemical variability to have a diverse range of activity. Additional file 1 shows 2D structures of the 18 mPTPB inhibitors of the congeneric series along with their IC_50_ and pIC_50_ values. After optimization and template based alignment of these compounds, descriptors representing steric, electrostatic and hydrophobic energies at all lattice points of the grid around the molecules were computed. Training and test sets were selected for 6-hydroxy-benzofuran-5-carboxylic acid derivatives using random data selection method. 75 % of the total compounds i.e., 13 molecules were selected for the training set and the rest comprised the test set. The two sets are considered appropriate if they follow unicolumn statistics i.e., maximum of the test set is less than maximum of training set and minimum of the test set is greater than of training set (Table 1). In this study, these conditions were fulfilled for an appropriate QSAR analysis [21].

**Table 1:** Unicolumn statistics for the training and the test set.

Column Name	Maximum	Minimum
Training Set	1.4200	-1.3400
Test Set	0.8900	-0.7100

**Figure 2:**
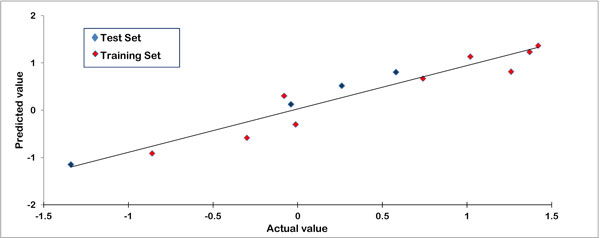
**Data fitness plot for the generated 3D QSAR model**.

**Figure 3:**
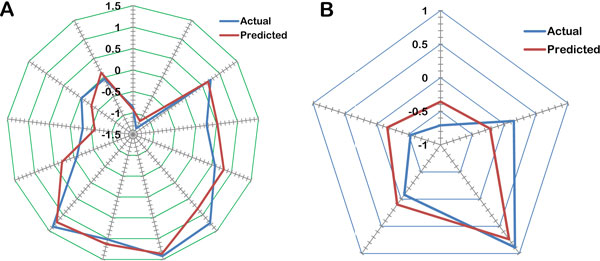
**Graph of actual and predicted biological activity for (a) training (b) test set**.

This made sure that the test set is interpolative and is derived from the min-max range of the training set. Stepwise forward algorithm in combination with principle component regression analysis (SW-PCA) was used to generate the model. The model developed by SW-PCA using random data selection method is shown in table 2. Table 3 shows the minimum recommended values for various statistical measures used to evaluate the model. Data fitness plot for the generated model is shown in Figure 2. The plot reflected its effectiveness as all the points lied close to the regression line. Figure 3a and 3b illustrates the radar plot of observed versus predicted biological activity values for both training and test sets of the developed model. The model can be used for external predictions as it has a high predictive correlation coefficient value of 0.8006.

**Table 2:** Statistics of the significant model generated using SW-PCA.

Parameters	Statistical Value
Training Set Size (n)	13
Test Set Size	5
Degree of freedom	11
r^2^	0.9170
q^2^	0.8920
F test	121.5937
r^2^ se	0.2586
q^2^ se	0.2950
pred_r^2^	0.8006
pred_r^2^se	0.3354

**Table 3:** Statistical measures with their minimum recommended values.

Statistical measures	Minimum recommended values
K	number of descriptors in a model (statistically n/5 descriptors in a model)
Df	degree of freedom (n-k-1) (higher is better)
q^2^	cross-validated r^2^ (>0.5)
q^2^se	Error term for q^2^
pred_r^2^	r^2^ for external test set (>0.5)
pred_r^2^se	Error term for pred_r^2^

**Figure 4:**
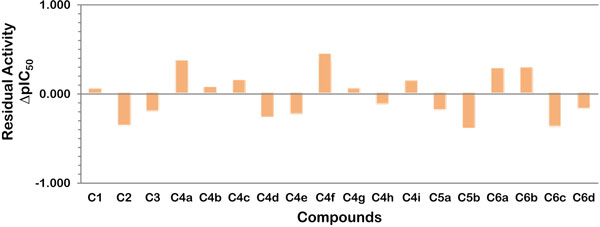
**Predicted residual activity of the derived compounds as evaluated by the QSAR model**.

**Figure 5:**
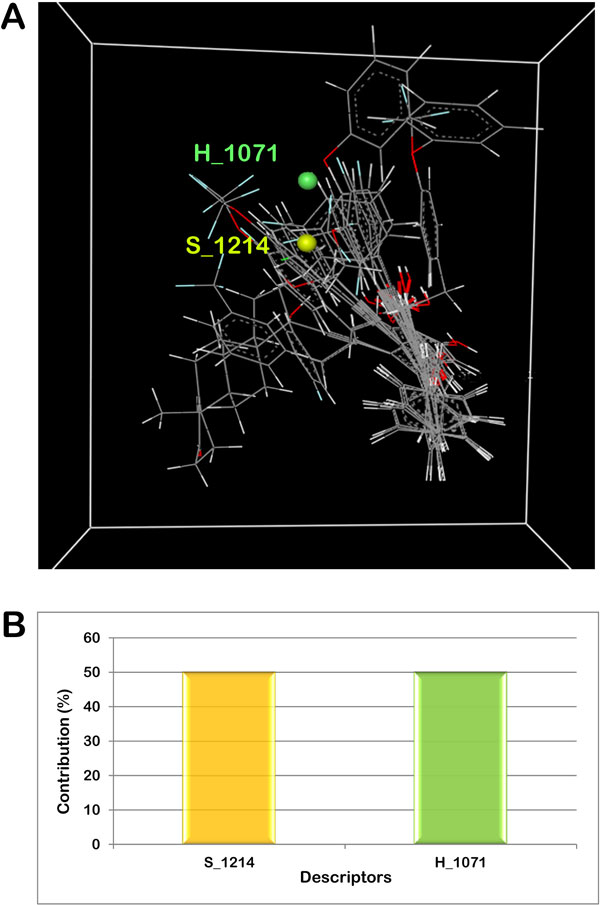
**(a) 3D-alignment of molecules with the important steric and hydrophobic points contributing to the biological activity of the ligands (b) Graph showing the contribution of molecular descriptors in controlling the activity of the inhibitors**.

The predicted biological residual activity (ΔpIC_50_= pIC_50__experimental- pIC_50__predicted) of benzofuran salicylic acid-based derivatives as evaluated by QSAR models is illustrated in Figure 4. The contour map (Figure 5a) provided further understanding of the relationship between structural features of 6-hydroxy-benzofuran-5-carboxylic acid derivatives and their activities which could be applied to design newer potential inhibitors of mPTPB.

### Interpretation of the built 3D QSAR model

The model had a good internal as well as external predictive power as indicated by the q^2^ value of 0.8920 and predicted r^2^ of 0.8006 respectively. It was observed that steric and hydrophobic descriptor at grid points, S_1214 and H_1071 play important role in imparting inhibitory activity against mPTPB. Figure 5b illustrates the contribution of these descriptors in controlling the activity of the inhibitors. The correlation between the molecular descriptors representing the physiochemical parameters of the ligands and their biological activity is given by the following equation:

The positive coefficient of S_1214 indicated that positive steric potential is preferred in that region and hence substitution of bulky groups will result in increased activity of the compounds.

Hydrophobic field descriptor (H_1071) also had a positive coefficient which suggested that the presence of more hydrophobic groups in this region would enhance the activity of the inhibitors. Presence of charged or polar groups around that grid point is not preferred for effective inhibitor design. The model provided a 3D fingerprint of the compounds which helped in developing a relationship of physiochemical parameters with structure and biological activity, making it capable of predicting activities of novel compounds. Thus, the 3D QSAR model generated can be used for fishing out novel natural compounds with inhibitory activity against mPTPB.

### Prediction of biological activity for a large dataset of natural compounds

A special subset of ZINC database consisting of 1,69,109 small molecules of natural origin was downloaded. The generated model had the statistical characteristics which proved it to be quite effective for external predictions. The generic prediction platform in 3D QSAR module of VlifeMDS was used to predict the activity values of these natural compounds. Table 4 lists the natural compounds which had the predicted pIC_50_ value greater than that of the most potent mPTPB inhibitor (comp10 with pIC_50_ of 1.42) of the congeneric series.

**Table 4:** List of natural chemical compounds with their pIC_50_ value predicted on the basis of the generated 3D QSAR model.

S.No.	ZINC IDs of natural compounds	pIC_50_ value
1.	ZINC08740008	1.800
2.	ZINC08765494	1.413
3.	ZINC12658654	1.904
4.	ZINC20610445	1.473
5.	ZINC38408096	1.605
6.	ZINC70664813	1.956
7.	ZINC70665093	1.677
8.	ZINC70673869	1.639
9.	ZINC72325052	1.624
10.	ZINC70665574	1.504
11.	ZINC59408720	1.503
12.	ZINC70698850	1.452
13.	ZINC02429300	1.623
14.	ZINC09130210	1.571
15.	ZINC67912863	2.198
16.	ZINC67912866	1.791
17.	ZINC70455089	1.771
18.	ZINC70454956	1.766
19.	ZINC59589328	1.528
20.	ZINC32787454	1.459
21.	ZINC04266010	1.700
22.	ZINC03845145	1.575
23.	ZINC68574485	1.550
24.	ZINC03978366	1.548

### Interaction analysis of the predicted natural compounds using *in silico* docking studies

**Figure 6:**
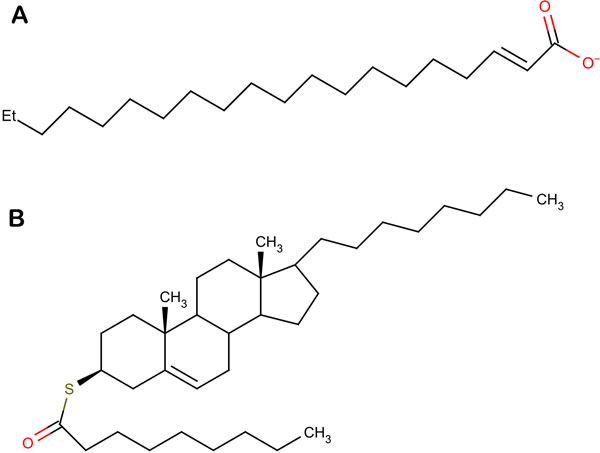
**Chemical structures of (a) first natural compound, ESA (b) second natural compound, DTP**.

**Figure 7:**
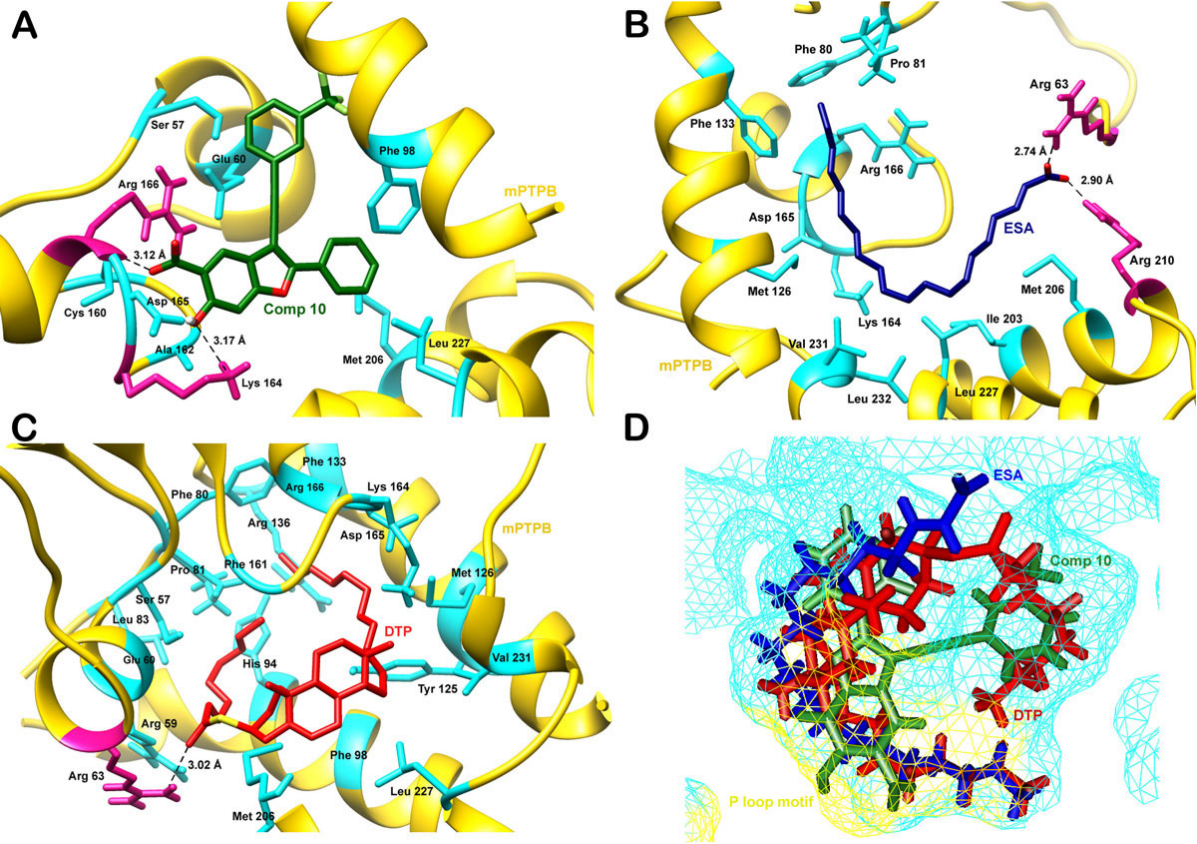
**(a) Molecular interactions between comp10 and mPTPB (b) ESA showing hydrogen bond (pink), hydrophobic and van der waal (cyan) interactions with mPTPB (c) Residues of mPTPB involved in hydrogen(pink), hydrophobic and van der waal (cyan) interactions with DTP (d) Relative position of all the three ligands in the cavity of mPTPB**.

The natural compounds with predicted pIC_50_ value greater than 1.42 were screened against the crystal structure of mPTPB using extra precision docking protocol of Glide. The two top scoring compounds, shown in Figures 6a and 6b, were studied to find their mode of interactions with the target protein. Interactions between comp10 (pIC_50_ = 1.42) and mPTPB were taken as reference (Figure 7a). Comp10 was forming 2 strong hydrogen bonds with Arg166 and Lys164 of mPTPB. It also showed hydrophobic interactions with various surrounding residues of the phosphatase namely Phe68, Ala162, Met206 and Leu227. The residues involved in van der waal interactions included Ser57, Glu60, Cys160 and Asp165. The first compound DELTA 2-trans Eicosenoic Acid had an activity value of 1.459. It was found forming hydrogen bond with residues Arg63 and Arg210, hydrophobic interactions with Phe80, Pro81, Met126, Phe133, Ile203, Met206, Leu227, Val231 and Leu232 and van der waal interactions with Lys164, Asp165 and Arg166 (Figure 7b).

The second compound S-((3S,10R,13R)-10,13-dimethyl-17-octyl-2,3,4,7,8,9,10,11,12,13,14,15,16,17-tetradecahydro-1H-cyclopenta[a]phenanthren-3-yl) nonanethioate also showed good binding affinity for mPTPB. It had an activity value of 1.677. Arg63 was involved in hydrogen bond formation while residues participating in hydrophobic interactions were Phe80, Pro81, Leu83, Phe98, Tyr125, Met126, Phe133, Arg136, Phe161, Met206, Val231 and Leu227 and van der waal interactions were Ser57, Glu60, His94, Lys164, Asp165 and Arg166 (Figure 7c). For ease in writing, these two screened compounds have been henceforth referred to as ESA and DTP. It was observed that all the three compounds had almost similar orientation or docking conformation, with ligands docked at the same position and interacting with the residues of P loop motif which forms the active site of mPTPB (Figure 7d). But the interactions of ESA (XP docking score = -7.62 kcal/mole) and DTP (XP docking score = -7.59 kcal/mole) with the mycobacterium phosphatase were stronger in comparison to comp10 (XP docking score = -6.75 kcal/mole). ESA was occupying more space in the cavity and was involved in more hydrophobic interactions, indicating a much stronger binding. DHP also showed intense binding by formation of hydrogen bond and multiple hydrophobic and van der waal interactions with the residues of the same cavity where comp10 fits in. Hence we can strongly suggest that these two compounds can potentially inhibit mPTPB enzymatic activity.

## Conclusion

A 3D QSAR model was generated for a congeneric series of 6-hydroxy-benzofuran-5-carboxylic acid derivatives having inhibitory activity against mPTPB. The model was generated using statistical method of principle component regression analysis in conjunction with stepwise variable selection method. The statistical measures r^2^, q^2^, F-test and standard error for the training set and the pred_r^2^ for the test set fulfilled the conditions for a model to be considered robust and predictive. The developed model was used to predict the activity values for a large set of natural compounds. The top scoring compounds were analyzed to find their mode of interactions with the mycobacterium phosphatase. We finally reported two natural compounds ESA and DTP which have high activity values of 1.459 and 1.677 respectively. They had a better affinity for mPTPB in comparison to the most potent compound of the congeneric series with pIC_50_ of 1.42, as observed from the docking score and the interaction pattern between these compounds and the mycobacterium protein. The present study provides substantial evidence for considering these natural compounds as prospective leads against tuberculosis having enhanced mycobacterium phosphatase inhibitory activity and low toxicity to human cells. Thus, 3D QSAR is an attractive discipline which not only provides graphical results that are often less attractive for scientific community but also has the ability to forecast the activity or potency of compounds being considered for inhibition of target protein. As QSAR approach already plays an important role in lead structure optimization, it is anticipated that it will soon become essential for handling large amount of data generated using combinatorial chemistry.

## Electronic supplementary material

Additional file 1: **This file includes the following table**. The list of novel mPTPB inhibitors along with their IC_50_ and pIC_50_ values (DOCX 339 KB)
