# Supplementary material for: Structural insights into mode of actions of novel natural Mycobacterium protein tyrosine phosphatase B inhibitors
Source: BMC Genomics. 2014 Jan 24;15(Suppl 1):S3. doi: 10.1186/1471-2164-15-S1-S3 (PMC4046716; doi:10.1186/1471-2164-15-S1-S3)
Supplement: Supplementary file 1 — Additional file 1: This file includes the following table. The list of novel mPTPB inhibitors along with their IC50 and pIC50 values (DOCX 339 KB) [file 12864_2014_5676_MOESM1_ESM.docx]

**Additional file 1**

**Additional file 1:** The list of novel mPTPB inhibitors along with their IC_50_ and pIC_50_ values

**S. No. Chemical structure IC_50_ (µM) pIC_50_**


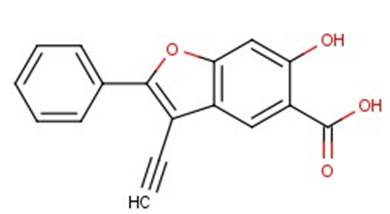


1. 7.300 -0.86


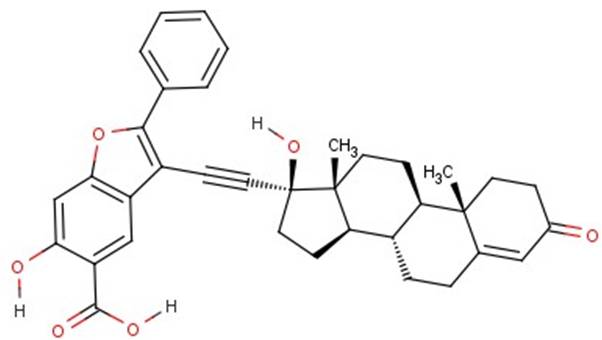


1. 5.090 -0.710


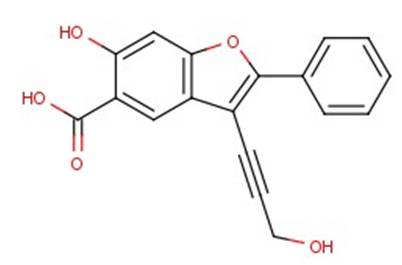


1. 22.000 -1.340


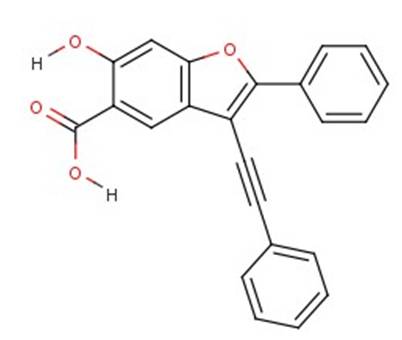


1. 0.700 0.150


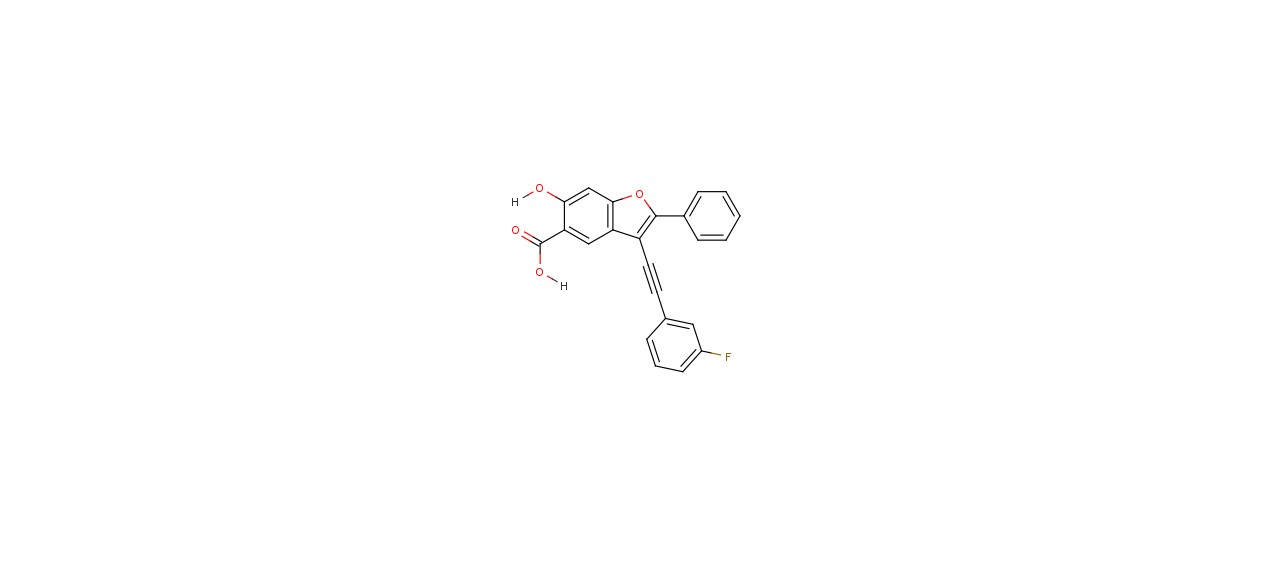


1. 0.180 0.740


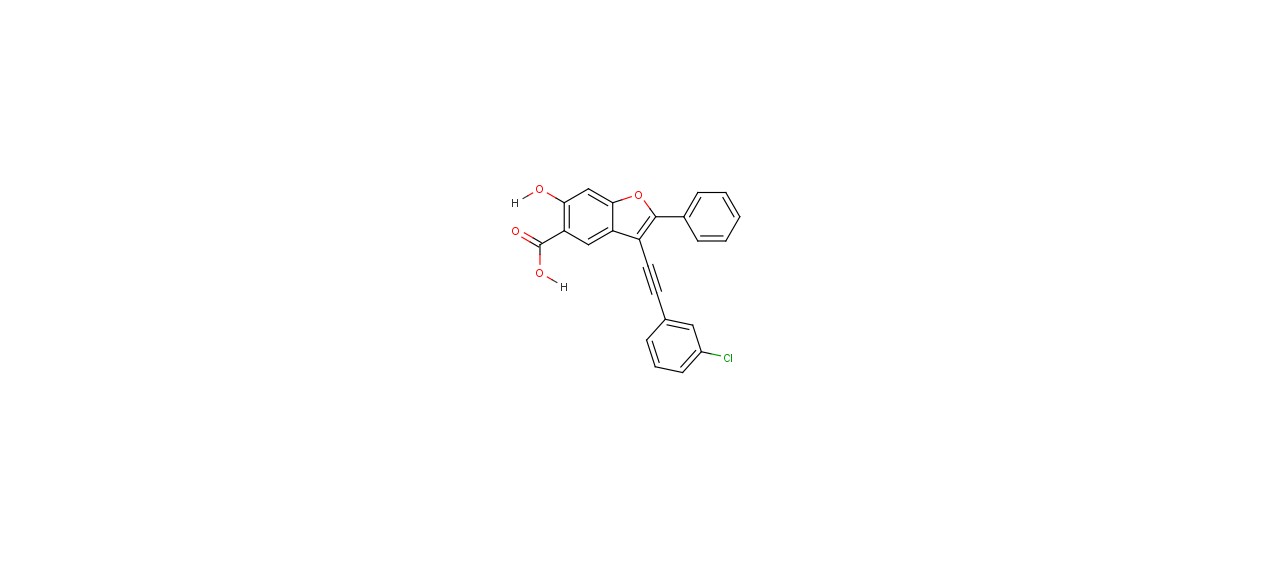


1. 0.130 0.890


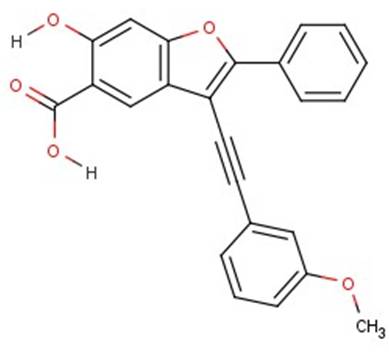


1. 0.540 0.260


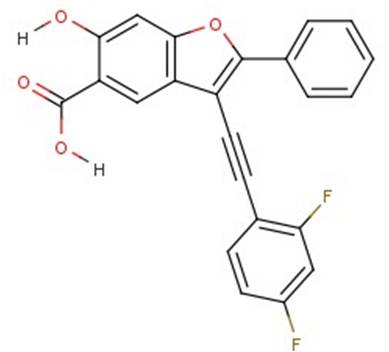


1. 0.260 0.580


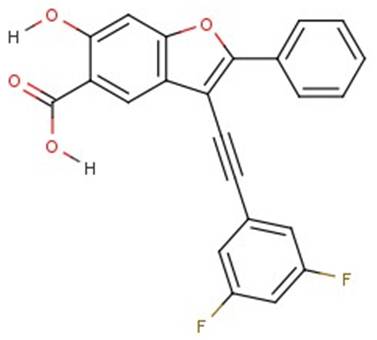


1. 0.540 1.260
2.
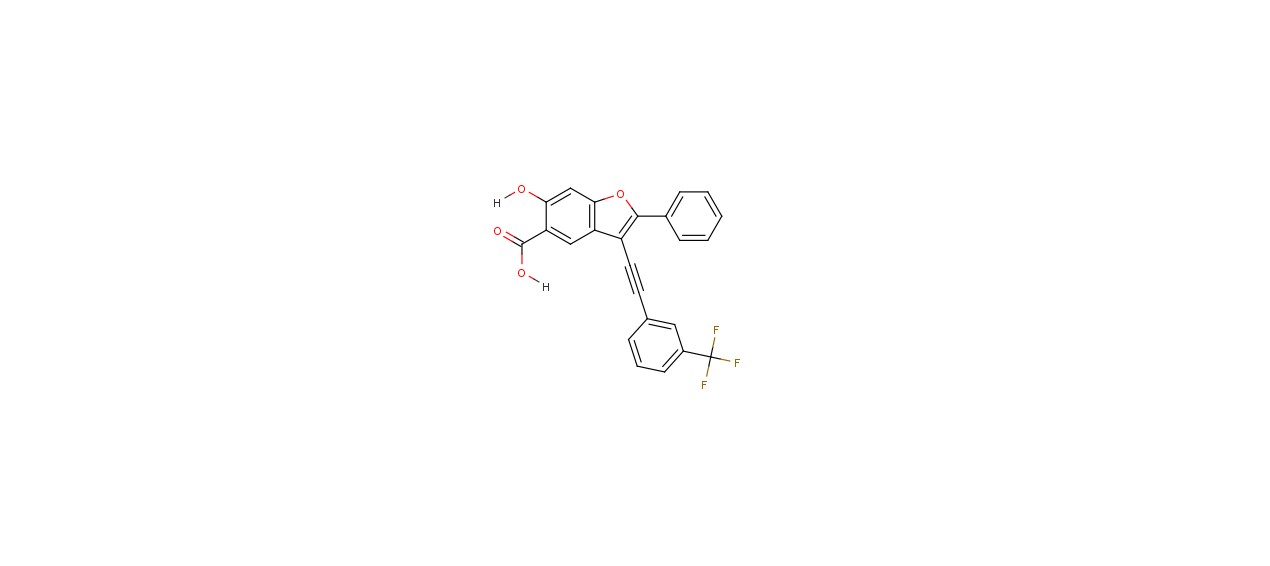
 0.038 1.420
3.
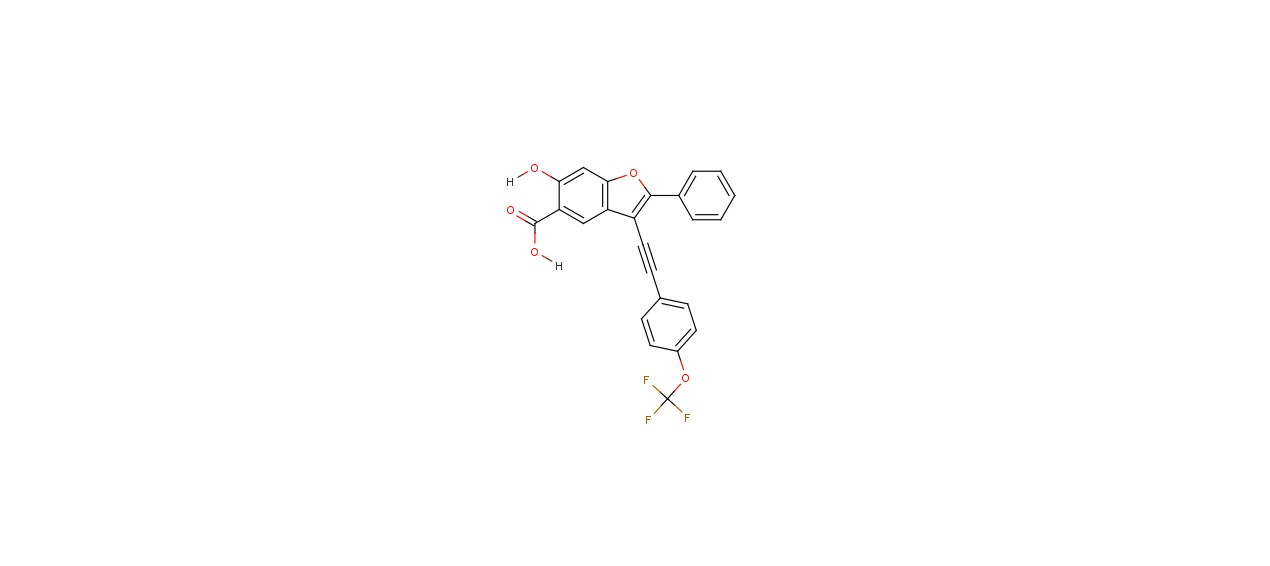
 0.095 1.020


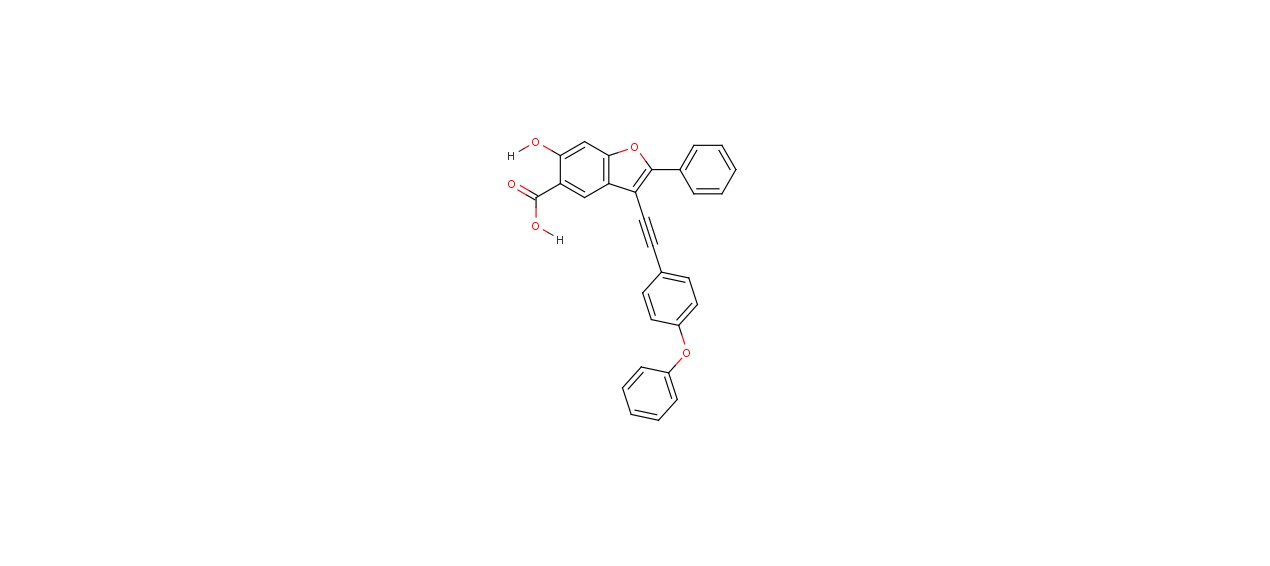


1. 0.043 1.370


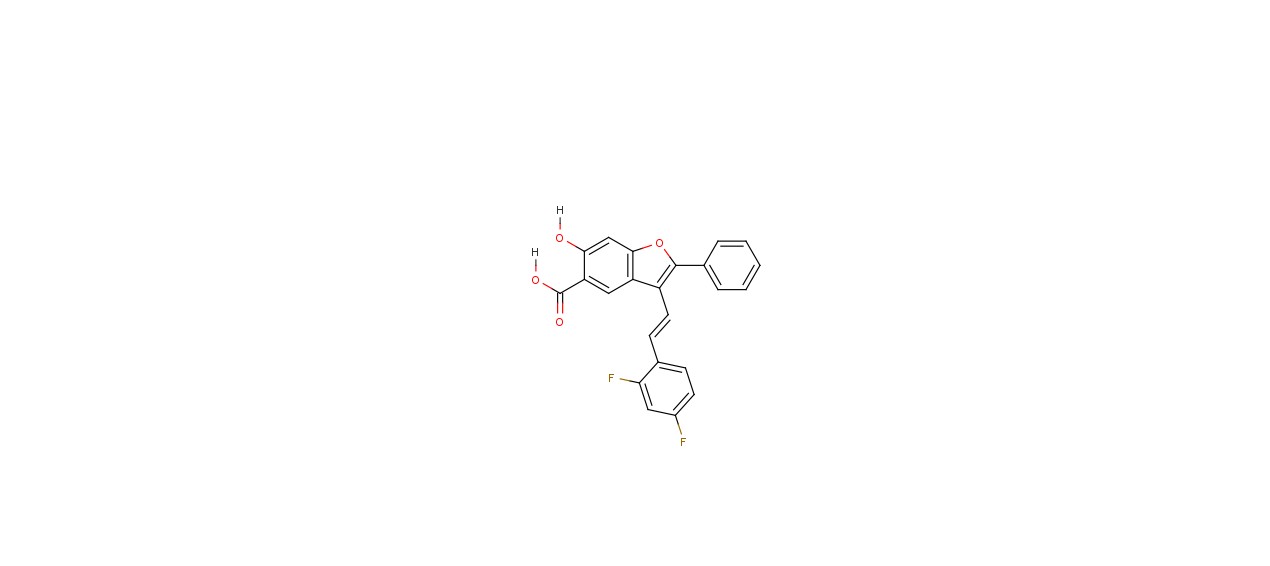


1. 1.200 -0.080


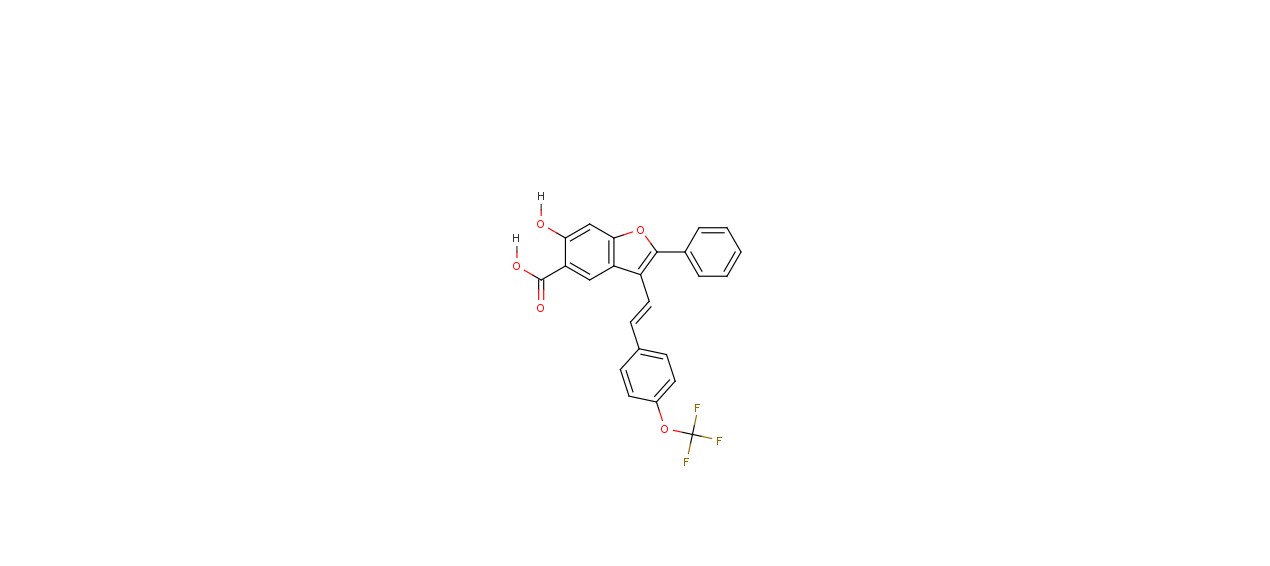


1. 1.200 -0.080
2.
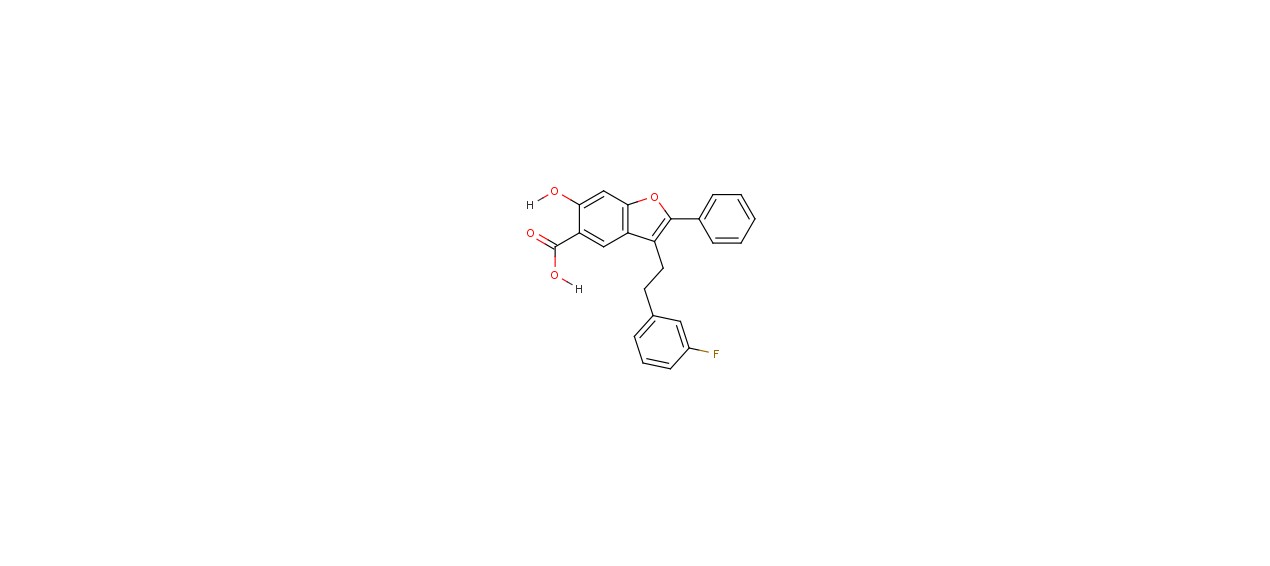
 2.000 -0.300


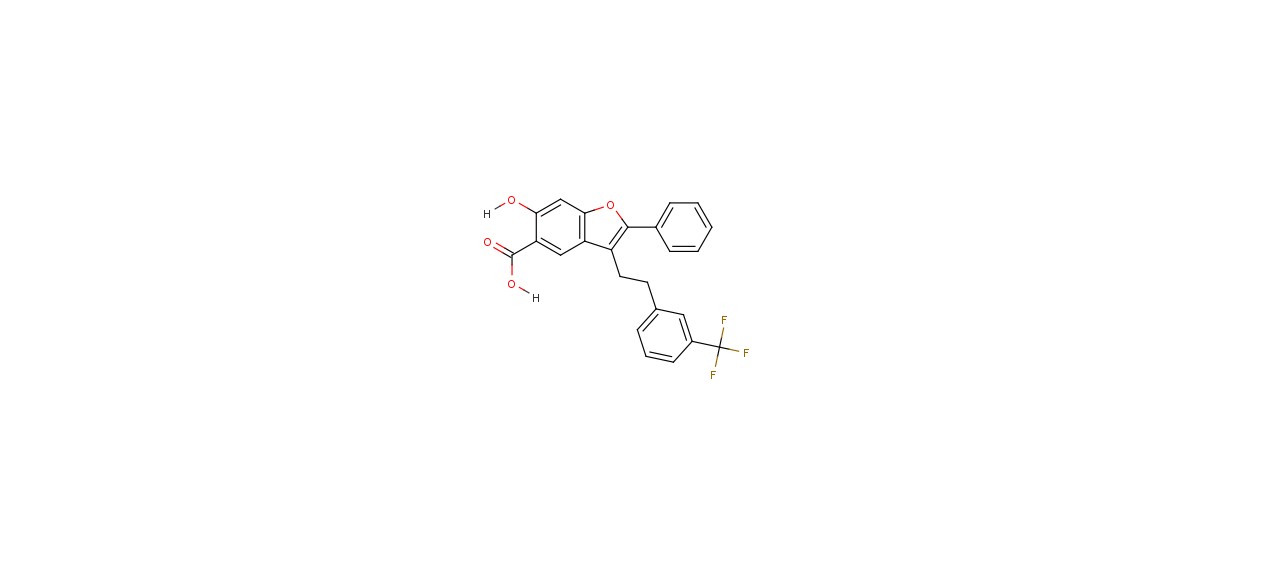


1. 1.030 -0.012


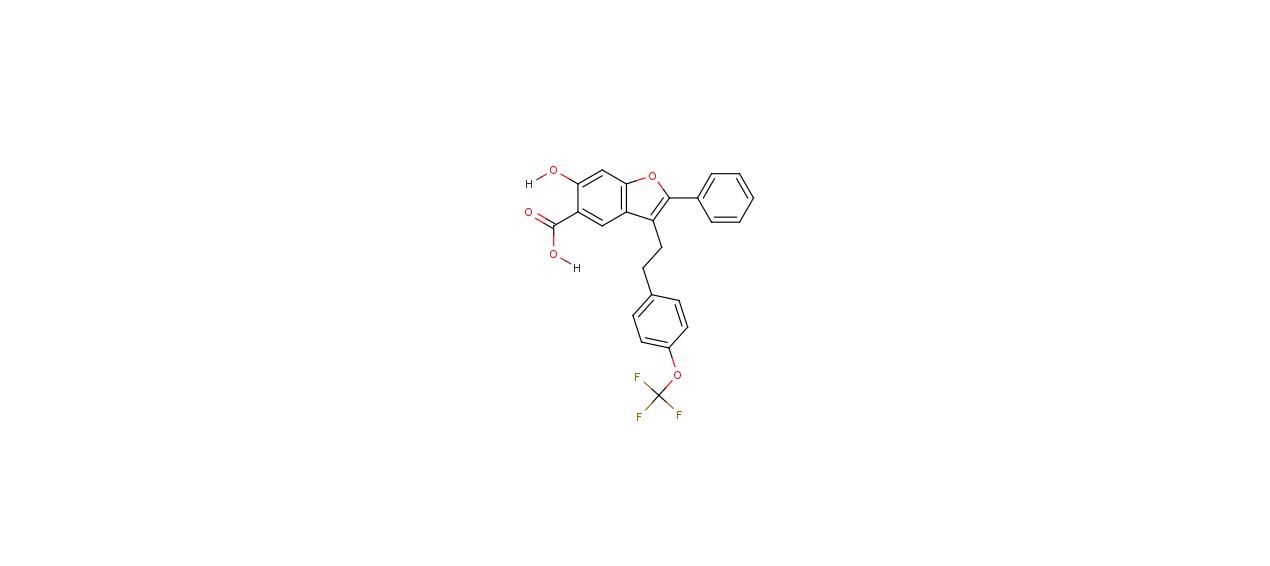


1. 3.400 -0.530


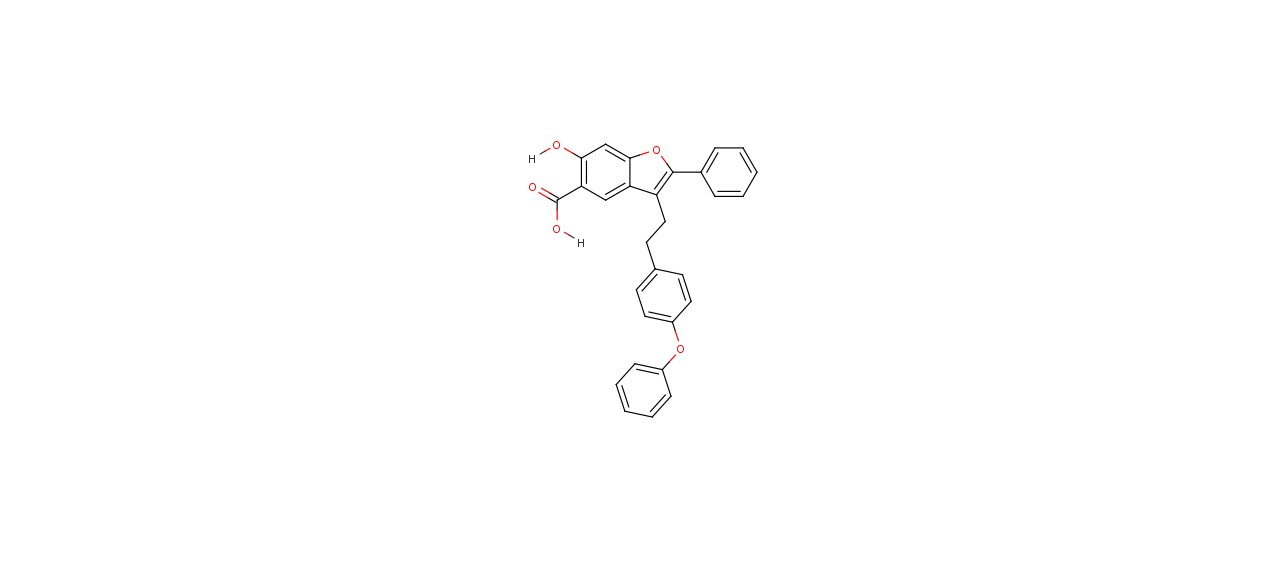


1. 1.100 -0.040
